# Genetic Variants of ABC and SLC Transporter Genes and Chronic Myeloid Leukaemia: Impact on Susceptibility and Prognosis

**DOI:** 10.3390/ijms23179815

**Published:** 2022-08-29

**Authors:** Raquel Alves, Ana Cristina Gonçalves, Joana Jorge, Gilberto Marques, André B. Ribeiro, Rita Tenreiro, Margarida Coucelo, Joana Diamond, Bárbara Oliveiros, Amélia Pereira, Paulo Freitas-Tavares, António M. Almeida, Ana Bela Sarmento-Ribeiro

**Affiliations:** 1 Laboratory of Oncobiology and Hematology (LOH), University Clinic of Hematology, Faculty of Medicine University of Coimbra (FMUC), University of Coimbra, 3000-548 Coimbra, Portugal; 2 Coimbra Institute for Clinical and Biomedical Research (iCBR)—Group of Environmental Genetics of Oncobiology (CIMAGO), Faculty of Medicine University of Coimbra (FMUC), University of Coimbra, 3000-548 Coimbra, Portugal; 3 Center for Innovative Biomedicine and Biotechnology (CIBB), 3004-504 Coimbra, Portugal; 4 Clinical Pathology Service, Centro Hospitalar e Universitário de Coimbra (CHUC), 3000-061 Coimbra, Portugal; 5 Hematology Service, Centro Hospitalar e Universitário de Coimbra (CHUC), 3000-061 Coimbra, Portugal; 6 Hemato-Oncology Laboratory, Instituto Português de Oncologia de Lisboa Francisco Gentil EPE, 1099-023 Lisbon, Portugal; 7 Laboratory of Biostatistics and Medical Informatics, Faculty of Medicine University of Coimbra (FMUC), University of Coimbra, 3000-548 Coimbra, Portugal; 8 Medicine Service, Hospital da Luz, 3020-479 Coimbra, Portugal; 9 Medicine Department, Hospital Distrital da Figueira da Foz, EPE, 3094-001 Figueira da Foz, Portugal; 10 Hospital da Luz Lisboa, 1500-650 Lisbon, Portugal; 11 Centro de Investigação Interdisciplinar em Saúde (CIIS), Faculdade de Medicina, Universidade Católica Portuguesa de Lisboa, 2635-631 Lisbon, Portugal

**Keywords:** CML, cancer predisposition, TKI resistance, drug transporters

## Abstract

Solute carrier (SLC) and ATP-binding cassette (ABC) transporters comprise a variety of proteins expressed on cell membranes responsible for intrusion or extrusion of substrates, respectively, including nutrients, xenobiotics, and chemotherapeutic agents. These transporters mediate the cellular disposition of tyrosine kinase inhibitors (TKIs), and their genetic variants could affect its function, potentially predisposing patients to chronic myeloid leukaemia (CML) and modulating treatment response. We explored the impact of genetic variability (single nucleotide variants—SNVs) of drug transporter genes (*ABCB1*, *ABCG2*, *SLC22A1*, and *SLC22A5*) on CML susceptibility, drug response, and *BCR-ABL1* mutation status. We genotyped 10 SNVs by tetra-primers-AMRS-PCR in 198 CML patients and 404 controls, and assessed their role in CML susceptibility and prognosis. We identified five SNVs associated with CML predisposition, with some variants increasing disease risk, including TT genotype *ABCB1* (rs1045642), and others showing a protective effect (GG genotype *SLC22A5* rs274558). We also observed different haplotypes and genotypic profiles associated with CML predisposition. Relating to drug response impact, we found that CML patients with the CC genotype (rs2231142 *ABCG2*) had an increased risk of TKI resistance (six-fold). Additionally, CML patients carrying the CG genotype (rs683369 *SLC22A1*) presented a 4.54-fold higher risk of *BCR-ABL1* mutations. Our results suggest that drug transporters’ SNVs might be involved in CML susceptibility and TKI response, and predict the risk of *BCR-ABL1* mutations, highlighting the impact that SNVs could have in therapeutic selection.

## 1. Introduction

Non-receptor tyrosine kinase inhibitors (TKIs) must enter cancer cells to exert their effect by interacting with their targets. However, approximately 40% of clinically available drugs are organic cations at physiological pH, where charged molecules have poor ability to diffuse freely across membranes [1]. Drug transport is mediated by membrane transporters responsible for influx, such as solute carrier (SLC) family members, and efflux, such as members of ATP-binding cassette (ABC) family. Under normal conditions, these transporters play an essential role in cell homeostasis, facilitating the uptake or extrusion of nutrients, metabolites, and other molecules [2].

BCR-ABL inhibitors, like imatinib, nilotinib and dasatinib, are TKIs used in the first-line treatment of CML, and can change the course of the disease [3]. The inter-individual variability of TKI responses among CML patients may be partly explained by pharmacogenomic factors [4,5]. The presence of polymorphic variants in genes that are actively involved in TKI transport may influence the pharmacokinetics and, consequently, the efficacy of these drugs [5,6]. Variations in genes that encode influx and efflux transporters, as well as in enzymes involved in drug metabolism, have been referred to as possible independent mechanisms of *BCR-ABL1* resistance [7,8]. The transport of BCR-ABL TKIs is mediated by the influx transporters OCT1 and OCTN2 (encoded by *SLC22A1* and *SLC22A5* genes) [9,10] or can occur by passive diffusion, as in the case of dasatinib and ponatinib [11,12]. In contrast, drug extrusion is performed by P-gP and BCRP (encoded by *ABCB1* and *ABCG2* genes) for all the approved TKIs [13,14,15]. These four genes are highly polymorphic, and some genetic variants result in altered activity of proteins (inducing changes on the binding sites of the substrate) or alterations ofexpression levels (changes in mRNA/protein stability or changes to localisation at membranes) [5,16]. Based on these factors, inherited genomic variants such as single nucleotide variants (SNVs), may influence drug availability and response. Furthermore, they could influence susceptibility to neoplasia, constituting a molecular marker for cancer risk, or a prognosis biomarker [16].

Given the importance of drug transporters in CML treatment, we evaluated the possible association of ABCB1, ABCG2, SLC22A1, and SLC22A5 genetic variants with disease prognosis (TKI response rates, number of TKIs required, and mutational state). Additionally, we investigated whether these genetic variants could predispose individuals to CML.

## 2. Results

### 2.1. Characteristics of the Study Group

This study enrolled 198 CML patients and 404 healthy control individuals (Table 1). The patient group was composed of 118 (59.6%) males and 80 (40.4%) females, with a median age of 54 years (range 15–86). The control group consisted of 236 (58.4%) males and 168 (41.6%) females, with a median age of 54 years (range 19–88). To avoid bias related to age and gender, we evaluated the differences in the demographic features. The absence of statistically significant differences for age (*p* = 0.738) and gender (*p* = 0.792) between the two groups confirmed adequate group matching.

In the CML group, most patients were diagnosed in chronic phase (95.0%, *n* = 188) and the majority (96.5%, *n* = 191) of these were treated with TKI (Table 1). In terms of response to treatment, 142 (74.3%) patients were classified as TKI responders while 49 (25.7%) were classified as resistant, based on the need for two or more lines of TKI. No statistically significant differences were found between these two sub-groups of patients according to demographic features (age, *p* = 0.374; gender, *p* = 0.315). Regarding first-line TKI treatment, almost all CML patients received imatinib as first-line TKI (95.3%), and in the TKI-resistant sub-group all were treated with imatinib up-front. In our cohort, 12 patients (6.3%) were exposed to three or more TKI lines during treatment and 22 (20.2%) developed *BCR-ABL1* mutations.

### 2.2. Allele and Genotype Distribution

We assessed the association of selected SNVs with CML development to infer the contribution of these variants to disease risk. We performed this analysis using allele [minor (m) allele compared to major (M) allele as reference] and genotype distribution. In the genotypic analysis, we applied four genetic models: (1) the codominant model (MCD)—where each genotype was compared with the homozygous major allele (mm or mM vs. MM), (2) the dominant model (MD)—minor allele carriers against major allele homozygous (mm + mM vs. MM), (3) the recessive model (MR)—minor allele homozygous compared to major alleles carriers (mm vs. MM + mM), and (4) the overdominant model (MOD)—heterozygous against homozygous individuals (mM vs. MM + mm).

Allele distribution in both groups of study is represented in Table 2. For the selected SNVs, our control population presented the same minor allele as described for Iberian populations in Spain, except for *SLC22A5* rs274558. In this SNV, the G allele was reported as the minor allele (MAF = 0.364), but in our population, the A allele was the minor allele (MAF = 0.450). *ABCG2* rs2231137 was the SNV with lowest MAF (0.066) and *SLC22A5* rs2631365 the highest (MAF = 0.499). Carriers of the T allele at *ABCB1* rs1045642 (OR = 1.483, 95%CI 1.154–1.906, *p* = 0.002) had a higher incidence of CML. In contrast, some alleles were significantly less frequent in the CML population, namely, the A allele of *ABCG2* rs2231142 (OR = 0.589, 95%CI 0.388–0.892, *p* = 0.012), the G allele of *SLC22A5* rs274558 (OR = 0.598, 95%CI 0.469–0.762, *p* < 0.001), and the C allele at rs2631365 of the same gene (OR = 0.682, 95%CI 0.534–0.869, *p* = 0.002).

We also observed significant associations between specific genotypes and CML development (Table 3). All genotypes among the study groups were in Hardy–Weinberg equilibrium (HWE). For *ABCB1* rs1045642, the carriers of one or two T alleles presented increased risk of CML development (MCD CT: OR = 1.469, 95%CI 1.017–2.121, *p* = 0.040/TT: OR = 2.373, 95%CI 1.343–4.193, *p* = 0.003; MR TT: OR = 1.929, 95%CI 1.134–3.28, *p* = 0.015), while the CC genotype showed a protective effect (MD: OR = 0.62, 95%CI 0.438–0.884, *p* = 0.008). In the same gene, the heterozygous genotype (CT) of rs1128503 was associated with lower CML susceptibility (MCD: OR = 0.640, 95%CI 0.432–0.948, *p* = 0.026). The AA genotype of *ABCG2* rs2231142 was not detected in CML patients, but individuals homozygous for the major allele (CC genotype) presented increased predisposition to CML (MD: OR = 1.601, 95%CI 1.034–2.480, *p* = 0.035). Furthermore, both SNVs evaluated on the *SLC22A5* gene had an impact on CML susceptibility. Individuals homozygous for major allele had higher risk of CML development [rs274558 AA genotype (MD: OR = 1.847, 95%CI 1.259–2.710, *p* = 0.002) and TT genotype at rs2631365 (MD: OR = 1.734, 95%CI 1.192–2.524, *p* = 0.004)].

For rs274558, the GG genotype carriers (MCD: OR = 0.342, 95%CI 0.207–0.566, *p* < 0.001) and carriers of the C allele in rs2631365 (MCD: TC OR = 0.630, 95%CI 0.425–0.934, *p* = 0.021 and CC OR = 0.448, 95%CI 0.267–0.752, *p* = 0.002) presented a protective effect against CML disease. Based on the genotype of SNVs that were associated with risk for CML (*ABCB1* rs1045642 TT, *ABCG2* rs2231142 CC, and *SLC22A5* rs274558 AA and rs2631365 TT), we analysed the risk of CML development according to the number of risk genotypes (Table 3). Only 6.1% (*n* = 12) of patients present no risk genotype, compared with 13.9% of controls. The presence of three or more risk genotypes conferred 8.667 times greater predisposition for CML disease (*n* = 39, *p* < 0.001). The SNVs evaluated in *ABCB1*, *SLC22A1*, and *SLC22A5* were in linkage disequilibrium in patients and also in control groups.

### 2.3. Haplotype and Genotypic Profiles Analysis

**Figure 1:**
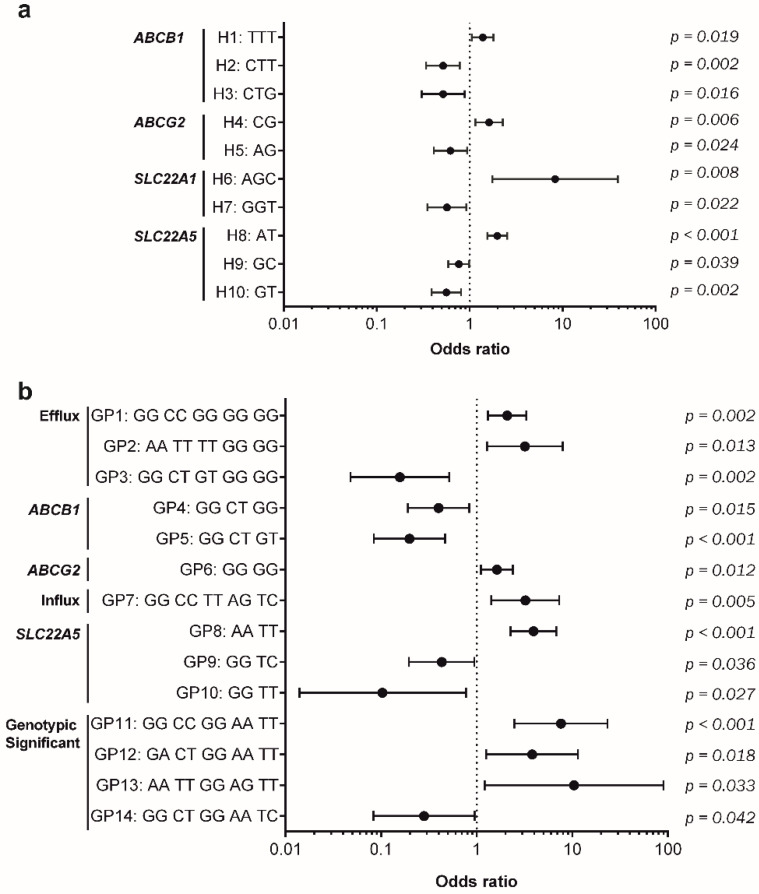
Haplotypes (H) and genotypic profiles (GP) significantly correlated with CML development; (**a**) the haplotypes and (**b**) the GPs positively correlated with CML susceptibility. The respective *p* value is shown for each analysis. Haplotype groups: *ABCB1*: rs1045642/rs1128503/rs2032582; *ABCG2*: rs2231142/rs2231137; *SLC22A1*: rs628031/rs683369/rs1867351; *SLC22A5*: rs274558/rs2631365. Genotypic profile categories: Efflux (*ABCB1*: rs1045642/rs1128503/rs2032582/*ABCG2*: rs2231142/rs2231137); Influx (*SLC22A1*: rs628031/rs683369/rs1867351/*SLC22A5*: rs274558/rs2631365); *ABCB1*: rs1045642/rs1128503/rs2032582; *ABCG2*: rs2231142/rs2231137; *SLC22A5*: rs274558/rs2631365; Genotypic Significant (*ABCB1*: rs1045642/rs1128503/*ABCG2*: rs2231142/*SLC22A5*: rs274558/rs2631365).

Studying several SNVs in the same gene allows analysis of haplotypes and their contribution to disease. In Figure 1a, we present the haplotypes identified in our population that correlated positively or negatively with CML development. Haplotype 6 (H6: AGC) for the *SLC22A1* gene (rs628031/rs683369/rs1867351) conferred about eight-fold higher risk (OR = 8.309, 95% CI 1.756–39.310, *p* = 0.008) of CML development compared to other haplotypes. In contrast, haplotypes H2 (CTT) and H3 (CTG) from *ABCB1* showed the highest protective effect (H2: OR = 0.517, 95%CI 0.341–0.784, *p* = 0.002; H3: OR = 0.518, 95%CI 0.304–0.884, *p* = 0.016).

Drug transport is a complex process involving multiple proteins, influx and efflux transporters, which affect oncogenesis and treatment response. To integrate the contribution of the multiple genetic variants studied in the identification of high-risk individuals, we performed a genotypic profile (GP) analysis. Using Arlequin software, GPs were inferred and grouped into eight categories: (1) Global profile which included 10 SNVs; (2) efflux profiles including five SNVs on ABC genes; (3) influx profiles including five SNVs of SLC transporters; (4–7) profiles for each gene (*ABCB1*, *ABCG2*, *SLC22A1*, and *SLC22A5*); and (8) significant genotypic profile with five SNVs significantly associated in the genotype distribution. We found 14 significant GPs associated with CML development (Figure 1b). GP1, GP2, and GP6 were related to efflux, accompanied by GP7 and GP8 related to influx, conferring increased predisposition to CML disease. Moreover, GP3 from the efflux category and GP10 of *SLC22A5* were the most protective profiles (OR = 0.157, 95% CI 0.048–0.517, *p* = 0.002; and OR = 0.103, 95% CI 0.014–0.774, *p* = 0.027, respectively). Of the significant genotypic category, GP13 and GP11 conferred approximately 10 and 8-fold higher risk of CML development, respectively, while GP14 was associated with a strong protective effect reducing CML susceptibility about four-fold (Figure 1b). Moreover, some GPs were only observed in patients’ groups, but, despite a significant OR, their 95%CI values were too diverse to be considered relevant.

### 2.4. Prognostic Value of Genetic Variants

**Figure 2:**
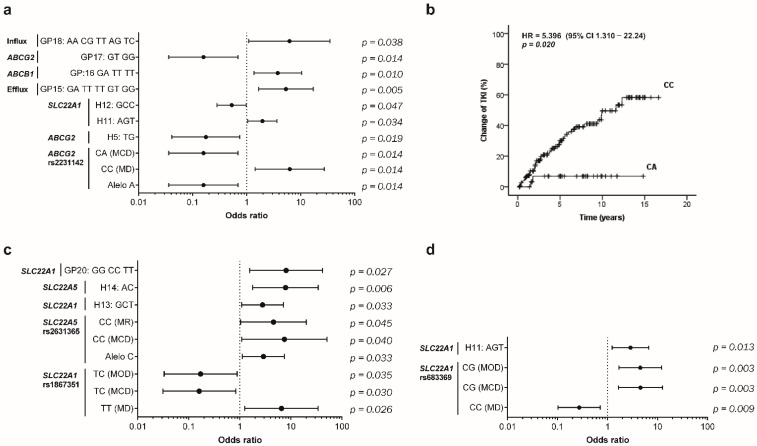
Prognostic impact of SNVs in influx and efflux transporters genes in CML patients. In (**a**) are the alleles, genotypes, haplotypes (H), and genotypic profiles (GP) significantly associated with TKI response in CML patients; (**b**) depicts the time to TKI change in CML patients according to *ABCG2* (rs2231142) genotypes. This analysis was performed by the Kaplan Meier–method, differences in survival were tested with the log-rank test, and hazard ratios (HR) with 95% confidence interval (CI) were calculated using the Cox proportional hazard model. In (**c**) are the alleles, genotypes, haplotypes, and GPs significantly associated with the number of TKIs needed by CML-resistant patients. In (**d**) are the alleles, genotype, haplotypes and GPs significantly associated with *BCR-ABL1* mutations in CML patients. The respective *p* value is shown for each analysis. Haplotypes groups: *ABCG2*: rs2231142/rs2231137; *SLC22A1*: rs628031/rs683369/rs1867351; *SLC22A5*: rs274558/rs2631365. Genotypic profile categories: Efflux (*ABCB1*: rs1045642/rs1128503/rs2032582/*ABCG2*: rs2231142/rs2231137); Influx (*SLC22A1*: rs628031/rs683369/rs1867351/*SLC22A5*: rs274558/rs2631365); *ABCB1*: rs1045642/rs1128503/rs2032582; *ABCG2*: rs2231142/rs2231137; *SLC22A1*: rs628031/rs683369/rs1867351. MD—model dominant; MR—model recessive; MOD—model overdominant; MCD—model co-dominant.

In addition to the correlation of genetic variants with the presence of CML, these variants may also be correlated with prognosis. One of the main issues in CML is the development of resistance to TKIs. To assess the association of selected SNVs with prognosis, we correlated them with TKI response, number of different TKIs needed, and development of *BCR-ABL1* mutations (Figure 2).

In our cohort, CML patients carrying the A allele of *ABCG2* rs2231142 had lower probability of developing TKI resistance, according to allele, genotype, haplotype (H5), and genotypic (GP17) profile frequencies, and their associated OR values (Figure 2a). In addition, a protective effect (OR = 0.456, 95%CI 0.243–0.856, *p =* 0.047) was also observed for the haplotype H12 (GCC) of the *SLC22A1* gene (rs628031/rs683369/rs1867351). Furthermore, CML patients with the CC genotype of *ABCG2* rs2231142 had a six-fold higher risk (95% CI 1.445–27.410, *p* < 0.014) of developing TKI resistance and five times less time before a second line of treatment (HR = 5.396, 95%CI 1.310–22.240, *p =* 0.020) in comparison with A-allele carriers (Figure 2b). Additionally, in the genotypic profile analysis we identified three GPs that are associated with a higher risk of resistance, GP15 of efflux (OR = 5.346, 95%CI 1.658–17.240, *p =* 0.005), GP16 of *ABCB1* (OR = 3.769, 95%CI 1.365–10.410, *p =* 0.010), and GP18 of influx transporters (OR = 6.222, 95%CI 1.103–35.120, *p =* 0.038).

Resistant patients may require more than two treatment lines (Figure 2c). We observed an association between SNVs in influx genes and the number of TKIs required by resistant patients. The TT genotype at rs1867351 *SLC22A1* and CC at rs2631365 *SLC22A5* were associated with the number of TKI lines of treatment (three or more TKIs: TT OR = 6.562, 95%CI 1.258–34.230, *p =* 0.026; CC: OR = 7.500, 95%CI 1.092–51.510, *p =* 0.040). The needed for multiple TKIs was particularly associated with GP20 of *SLC22A1* (OR = 8.095, 95%CI 1.560–42.020, *p =* 0.027). The development of mutations in *BCR-ABL1* is a well-documented mechanism of TKI resistance and has an important impact on prognosis. In our cohort, the CG genotype of rs683369 *SLC22A1* was associated with the development of *BCR-ABL1* mutations (OR = 4.565, 95%CI 1.660–12.550, *p =* 0.003) while fewer mutations occurred in those with the CC genotype (OR = 0.271, 95%CI 0.102–0.721, *p =* 0.009) (Figure 2d). We could not find a significant association between selected SNVs and overall survival and progression.

## 3. Discussion

Each genetic variant leads to different gene or protein expression consequences, which ultimately modulates protein activity [16]. The impact of these variants on normal healthy cells and cancer cells differs according to their cellular context [17]. Several SNVs of influx and efflux drug transporter genes have been described, that result in lower gene expression, incorrect cellular localisation, or altered protein activity [2]. In healthy cells, these variants may lead to higher exposure to xenobiotics, metabolites, and toxins, changing the cellular homeostasis and promoting carcinogenesis [2,17,18]. The unbalanced transport may create a more pro-oncogenic environment leading to higher genetic instability, especially relevant in highly proliferative regions, such as hematopoietic tissues. In contrast, when we analyse the impact of these variants on cancer cells, it appears the low activity of efflux transporters, which export anticancer drugs, may improve drug efficacy and thus be associated with better prognosis [8]. Based on this, SNVs may have a crucial role not only in disease severity but also in predisposition [7,19]. All the genetic variants we studied have been onserved to induce lower gene expression or reduced protein activity [16,20,21]. We have shown that three SNVs correlated positively with the development of CML, three correlated with prognosis, and two correlated with CML development and prognosis.

ABC transporter genes are highly polymorphic, and multiple variants have been associated with oncogenesis and prognosis. P-glycoprotein, codified by *ABCB1*, plays a vital role in cell detoxification, in the transport of substrates across cells and tissues, as well as in the pharmacokinetics of anticancer drugs and their intracellular concentrations [6,19]. We observed that the T allele and TT genotype of rs1045642 *ABCB1* increased about 1.5- and 2.4-fold the risk of CML developing, respectively. Corroborating our results, other groups have found an association between the T allele and the respective genotypes homozygous to leukemogenesis [22,23,24,25,26] and other cancers [27]. Another synonymous variant of *ABCB1*, rs1128503, has also been linked to risk for leukaemia in individuals carrying the TT genotype [28,29]. On the contrary, we found that heterozygotes had 1.6 times lower incidences of CML. rs2032582 is one of the most studied SNVs of *ABCB1*. In contrast to descriptions in other tumours, we did not find any association of this variant with CML [27,30]. This has also been reported by Vivona et al. [31] and Yaya et al. [28] in different populations. However, we found that the TTT haplotype (H1) that comprises the three studied variants of *ABCB1* was associated with CML development. Potocnik et al. [30] and Wu et al. [27] observed the same correlation between this haplotype and colorectal cancer and breast cancer. ABCG2, which belongs to the same family of transporters, has been implicated in stem-cell protection and associated with some cancers [32]. The most studied variants, rs2231142 and rs2231137, present high variation in frequency according to the ethnicity of populations, being more frequent in Asians than in Caucasians. In our study, these were the two variants with the lowest MAF, but even so, rs2231142 was shown to be positively correlated with CML susceptibility. While the A allele conferred protection (OR = 0.589), the CC genotype was associated with a 1.6 times higher risk of CML development. The relationship between common *ABCG2* variants and cancer risk is complex and controversial, and the main differences seem to be dependent on population and cancer type [33]. In accordance with our results, a protective effect in genotypes with allele A was reported in European patients with B-cell non-Hodgkin lymphoma and chronic lymphocytic leukaemia [34]. However, Wu et al. described A allele and AG haplotype as risk factors for breast cancer in Chinese populations [35]. Our results showed that the AG haplotype (H5) conferred a protective effect while CG (H4) increased the risk of CML in this Portuguese population.

*SLC22A1* and *SLC22A5* encode OCT1 and OCTN2 influx drug transporters, respectively, whose function has been associated with responses to multiple drugs [6,19]. OCT1 is the principal hepatic drug uptake protein, and SNVs on this protein contribute to different responses to metformin and also affect codeine and morphine toxicities [36,37]. Moreover, variants in *SLC22A5* that cause loss of OCTN2 function have been associated with primary systematic carnitine deficiency [20] and Crohn’s disease [38]. The link between cancer predisposition and genetic variants of *SLC22A1* and *SLC22A5* is poorly explored. In our population, we observed significant associations between CML development and the two studied variants of *SLC22A5*. The G allele of the rs274558 variant showed a protective effect (OR = 0.598), more pronounced in individuals homozygous to the G allele who showed almost three times less (OR = 0.342) predisposition to CML, while the AA genotype increased the risk for CML 1.847 times. A similar pattern was observed for rs2631365, where the minor allele (C) showed a protective effect and the AG haplotype (H8) induced a higher risk of CML development. Additionally, the AGC haplotype (H6) of *SLC22A1* conferred the highest risk of CML (OR = 8.309).

Looking for variants individually is an important approach for understanding their individual impact, but it is also relevant to carry out a more complex analysis, which considers the combination of several SNVs. We counted the number of risk genotypes in each individual and observed that individuals carrying three or more risk genotypes have eight times more propensity to develop CML. Another possible way to study these complex interactions is by tracing genetic profiles correlated with disease predisposition. Zhang et al. [39] indicated that patients with CC and TT genotypes for *ABCG2* rs2231142 and rs1045642 of *ABCB1*, respectively, had a higher risk of CML development. In our population, the efflux profile category allowed us to identify one protective profile (GP3) and one risk profile (GP2) that include TT homozygosity for all *ABCB1* SNVs. In case of the influx profile category, only GP7 was relevant and associated with disease risk. Looking only at *SLC22A5* variants, the GP10 profile was associated with the lowest OR (0.103) of CML development. We also identified a genetic profile that conferred 10 times more risk of CML (GP13), which included the same SNVs of efflux transporters described by Zhang et al. [39] with additional SNVs of influx transporters.

TKI efficacy is highly dependent on cellular drug concentration to effectively block BCR-ABL activity, and low TKI concentrations have already been associated, at least in part, with reduced expression or activity of SLC transporters and/or increased expression or activity of ABC transporters [40,41]. The SNVs studied here, which are associated with altered expression and activity, have a potential role in response to therapy [42,43]. Recently Rajamani et al. showed the influences of *ABCB1* polymorphisms and plasma imatinib levels on early molecular response and failure-free survival [41]. Despite frequently having been associated with response by other authors [5,8,44,45], P-gP SNVs did not present a prognostic impact in our study. According to the literature, rs2231142 of *ABCG2* results in loss of function mainly by altering protein folding [45,46]. In our study, this SNV presented a correlation with CML susceptibility and drug response, and a significant transporter reduction has been linked to the A allele [18]. The CC genotype was associated with TKI resistance, whereas the CA was associated with good TKI response. In the same line, the AG haplotype (H5) was also correlated with good response to therapy. These findings reinforced those reported by other groups, where the A allele was associated with higher rates of molecular response [8,14,20], and the G allele of rs2231137 was associated with favourable therapeutic response [47]. Based on this, patients with the CC genotype had a 5.4 times higher chance of needin a change in TKI therapy, compared with the CA genotype (Figure 2b). The haplotype H11 of *SLC22A1* was significantly associated with TKI resistance, in concordance with Cargnin et al. who described a lower rate of MMR with the A allele of rs628031 and G allele of rs683369 [48]. Influx GP18 conferred a 6.2 times higher risk of resistance, while the GP17 of *ABCG2* correlated with a similar good response (6.3 times higher). In our study, genetic variants of the SLC family were highly associated with the number of TKIs needed after first line failure, and with *BCR-ABL1* mutation status. The rs2631365 of *SLC22A5* revealed an opposite effect on susceptibility and disease prognosis. While the C allele was protective of CML development in resistant patients, this allele was also positively associated with the requirement of more than two lines of TKIs. Other SNVs on this gene have been previously associated with time to progression in unresectable gastrointestinal stromal tumour (GIST) under TKI treatment, and with adverse TKI effects [49,50]. Moreover, the G allele in rs683369 of *SLC22A1* has previously been associated with lower MMR and high risk of resistance, due to low expression of OCT1 and consequent lower TKI uptake [7,20,51]. This variant correlates with mutational status in our population, where heterozygous (CG) CML patients presented 4.5 more risk of developing *BCR-ABL1* mutations. In addition to its correlation with resistance, the haplotype H11 was identified as a risk factor for development of *BCR-ABL1* mutations. This fact could be explained by exposure to subtherapeutic TKI concentrations, in consequence of these *SLC22A1* variants [52]. Lower drug uptake or high drug extrusion may create a favourable environment for the acquisition of other mechanisms of resistance, such as *BCR-ABL1* mutations [53].

The relevance of polymorphism on predisposition to disease or on prognosis has frequently been controversial due to inconsistent results. Sample size limitation, different frequencies according to patient ethnicity, treatment protocols, and response criteria are some factors that could explain the high heterogeneity between studies [19,44]. In addition to the factors listed, other mechanisms could also be relevant to explain controversial results, such as epigenetic mechanisms, namely DNA methylation and microRNA regulation, or post-translational modifications [40]. Even so, the identification of important SNVs profiles can have a high clinical impact on therapeutic selection. This tailored approach could improve response rates and avoid resistance cases, for instance allowing the use of dasatinib, which passive diffuses in cells, in patients with low influx-transporter activity or expression. In the same line, knowing patients’ SNVs information for variants associated with a higher risk of *BCR-ABL1* point mutations (as in the case of rs683369 *SLC22A1*) could justify early mutation screening and a quicker adaptation in treatment strategy. Moreover, patients with the CC genotype of rs2231142 ABCG2 will benefit from a shorter follow-up time since they present a higher risk of needing an early TKI switch, allowing early modification of the therapeutic protocol. These adjustments may enhance patients’ quality of life and consequently reduce the overload of healthcare systems.

## 4. Materials and Methods

### 4.1. Study Population

We performed a case–control study that enrolled 198 CML patients and 404 control individuals. Participants were recruited in three hospitals—“Centro Hospitalar e Universitário de Coimbra, EPE (CHUC, EPE)”, “Hospital Distrital da Figueira da Foz, EPE (HDFF, EPE)”, and “Instituto Português de Oncologia de Lisboa (IPO-Lisboa, EPE)”—between September 2014 and August 2017. Patients were diagnosed according to World Health Organization classification, and treatment response criteria were defined based on European Leukemia Net (ELN) guidelines [54]. At the same time, controls were selected in participant hospitals from healthy blood donors and individuals with no history of cancer. Cases and controls were matched by gender and age (±5 years) to minimise the effect of confounders. Demographic and clinical characteristics of patients and controls are summarised in Table 1. The patient group was subdivided into TKI responders (*n* = 142) or resistant (*n* = 49) according to therapeutic response, for prognostic analysis (Table 1). Excluded from the study were patients intolerant to therapy, to avoid confounder effects. The study was conducted according to the Helsinki Declaration, and all participants provided written informed consent for participation before enrolment. All research procedures were approved by the Ethics Committee of the Faculty of Medicine (University of Coimbra, Portugal) (ref. CE-014/2014).

### 4.2. Genes and SNVs Selection

The selection of candidate genes was based on previously reported involvement in TKI influx and efflux. *ABCB1*, *ABCG2*, *SLC22A1*, and *SLC22A5* were the genes selected as involved in TKI transport [9,20]. Ten SNVs (two or more per gene) were selected according to the following criteria: (1) Localisation in a coding region; (2) minor allele frequency (MAF) ≥ 5% in Caucasian Iberian population, available in public databases; and (3) known or promising relevance to cancer predisposition and response to therapy. The characterisation of selected SNVs is detailed in Supporting Information Table S1.

### 4.3. Genotyping

Genomic DNA was extracted from whole blood samples, collected into EDTA tubes, using NZYol reagent (NZYtech, Lisbon, Portugal) according to manufacturer protocol. Quality and quantity of DNA were determined using a NanoDrop ND-1000 spectrophotometer (NanoDrop Technologies, Wilmington, NC, USA), and 100 ng of DNA was used in each genotyping reaction. All SNVs were genotyped through tetra-primer-ARMS-PCR assays with primers designed using BatchPrimer3, version 1.0 (USDA-ARS, Albany, CA, USA) (http://probes.pw.usda.gov/batchprimer3/ (accessed on 20 September 2014)) [55]. PCR reaction conditions and the resulting products are described in Supporting Information Table S2. Results were confirmed first by direct sequencing, and the samples were found to contain the three possible genotypes that were used as positive controls in each genotype assay. To assess genotyping accuracy, genotyping was repeated in approximately 10% of the total samples.

### 4.4. Statistical Analysis

Differences of confounding variables (age and gender) between groups were assessed by nonparametric Mann–Whitney U test and Fisher’s exact test. Allele and genotype frequencies were determined by direct counting. Hardy–Weinberg equilibrium (HWE) in the study groups and the genotypic profile (GP) frequencies were determined by Arlequin software, version 3.5.1.2 (CMPG, University of Bern, Bern, Switzerland) [56]. Fisher’s exact test was calculated in order to compare the allele frequencies among groups, using GraphPad Prism, version 5.0 (GraphPad Software, San Diego, CA, USA). The associations between genotype, haplotype, and GP, and CML or clinical characteristics were analysed by calculating the odds ratio (OR) and 95% confidence interval (CI), applying unconditioned logistic regression, with SPSS, version 23.0 (IBM, NY, USA). The Kaplan–Meier method was carried out to estimate the time to change in TKI for patients dichotomised according to their genotypes using SPSS, and differences were tested through a log-rank statistic. The hazard ratio (HR) and its 95% CI were calculated using the Cox proportional hazard model. All statistical analyses were two-sided, and *p* < 0.05 was considered statistically significant.

## 5. Conclusions

In conclusion, we have highlighted the impact of genetic variants in drug transporter genes on predisposition and prognosis of CML. The findings on SNVs may be a useful tool for understanding inter-individual variability and improving therapy decisions, including treatment selection.

## Figures and Tables

**Table 1:** Demographic and clinical characteristics of patients and controls.

Characteristics	CML						Controls (*n* = 404)	
	*All Patients* (*n* = 198)		*TKI Responder* (*n* = 142)		*TKI Resistant* (*n* = 49)			
**Demographic features**								
Gender (%)								
Male	118	(59.6)	80	(56.3)	32	(65.3)	236	(58.4)
Female	80	(40.4)	62	(43.7)	17	(34.7)	168	(41.6)
Age (years)								
Median	54		54		51		54	
Range	15–86		15–86		18–79		19–88	
**Clinical features**								
Phase of Disease								
Chronic Phase (%)	188	(95.0)	135	(95.1)	46	(93.9)		
Accelerate Phase (%)	5	(2.5)	5	(3.5)	–	–		
Blast Crisis (%)	5	(2.5)	2	(1.4)	3	(6.1)		
Scoring Systems								
Sokal Score	(*n =* *144*)		(*n =* *107*)		(*n =* *33*)			
Low Risk (%)	79	(54.9)	61	(57.0)	16	(48.5)		
Intermediate Risk (%)	47	(32.6)	33	(30.8)	12	(36.4)		
High Risk (%)	18	(12.5)	13	(12.2)	5	(15.1)		
Euro Score	(*n =* *144*)		(*n =* *107*)		(*n =* *33*)			
Low Risk (%)	106	(73.6)	81	(75.7)	21	(63.7)		
Intermediate Risk (%)	32	(22.2)	21	(19.6)	11	(33.3)		
High Risk (%)	6	(4.2)	6	(4.7)	1	(3.0)		
EUTOS Score	(*n =* *142*)		(*n =* *106*)		(*n =* *32*)			
Low Risk (%)	125	(88.0)	94	(88.7)	27	(84.4)		
High Risk (%)	17	(12.0)	12	(11.3)	5	(15.6)		
Treatment	(*n =* *198*)		(*n =* *142*)		(*n =* *49*)			
TKI (%)	191	(88.0)	142	(100.0)	49	(100.0)		
Other (%)	7	(12.0)	–	–	–	–		
First-line TKI	(*n =* *191*)		(*n =* *142*)		(*n =* *49*)			
Imatinib (%)	182	(95.3)	133	(93.7)	49	(100.0)		
Other TKI (%)	9	(4.7)	9	(6.3)	–	–		
Number of TKIs during treatment	(*n =* *191*)		(*n =* *142*)		(*n =* *49*)			
1 TKI (%)	142	(74.3)	142	(100.0)	–	–		
2 TKIs (%)	37	(19.4)	–	–	37	(75.5)		
≥3 TKIs (%)	12	(6.3)	–	–	12	(24.5)		
Mutations on *BCR-ABL1*	(*n =* *104*)		(*n =* *69*)		(*n =* *35*)			
Present (%)	22	(21.2)	11	(15.9)	11	(31.4)		
Absence (%)	82	(78.8)	58	(84.1)	24	(68.6)		

CML: chronic myeloid leukaemia; TKI: tyrosine kinase inhibitor.

**Table 2:** Allele distribution of selected SNVs in CML and controls, and its association with risk of CML.

Gene	dbSNP	Minor Allele ^‡^	CML			Controls
			MAF	OR (95% CI)	*p*-Value	MAF
*ABCB1*	rs1045642	T	0.396	**1.483 (1.154–1.906)**	**0.002**	0.307
	rs1128503	T	0.432	0.873 (0.685–1.113)	0.295	0.465
	rs2032582	T	0.402	1.034 (0.809–1.322)	0.802	0.394
*ABCG2*	rs2231142	A	0.081	**0.589 (0.388–0.892)**	**0.012**	0.130
	rs2231137	A	0.043	0.639 (0.365–1.190)	0.149	0.066
*SLC22A1*	rs628031	A	0.348	1.187 (0.919–1.532)	0.190	0.311
	rs683369	G	0.225	0.751 (0.567–0.996)	0.050	0.278
	rs1867351	C	0.328	1.206 (0.931–1.563)	0.161	0.288
*SLC22A5*	rs274558	G	0.422	**0.598 (0.469–0.762)**	**<0.001**	0.450 (A)
	rs2631365	C	0.407	**0.682 (0.534–0.869)**	**0.002**	0.499

^‡^ Minor allele according 1000 Genome database (Caucasians/European/Iberian population in Spain). Bold indicates statistically significant association. MAF: minor allele frequency; OR: odds ratio; CI: confidence interval; CML: chronic myeloid leukaemia.

**Table 3:** Significant genotype distribution of selected SNVs in CML and controls, and its association with risk to CML.

Gene: dbSNP	CML				Controls	
	*n*	%	OR (95% CI)	*p*-Value	*n*	%
***ABCB1:* rs1045642**						
CC	70	35.4	Ref.		189	46.8
CT	99	50.0	**1.469 (1.017–2.121)**	**0.040**	182	45.0
TT	29	14.6	**2.373 (1.343–4.193)**	**0.003**	33	8.2
CC (MD)			**0.622 (0.438–0.884)**	**0.008**		
TT (MR)			**1.929 (1.134–3.281)**	**0.015**		
CT (MOD)			1.220 (0.868–1.715)	0.253		
***ABCB1:* rs1128503**						
CC	67	33.8	Ref.		106	26.2
CT	89	45.0	**0.640 (0.432–0.948)**	**0.026**	220	54.5
TT	42	21.2	0.852 (0.525–1.382)	0.516	78	19.3
CC (MD)			1.438 (0.995–2.079)	0.053		
TT (MR)			1.125 (0.739–1.714)	0.583		
CT (MOD)			**0.683 (0.485–0.961)**	**0.029**		
***ABCG2:* rs2231142**						
CC	165	83.3	Ref.		306	75.7
CA	33	16.7	0.680 (0.437–1.057)	0.087	90	22.3
AA	0	0.0	–	–	8	2.0
CC (MD)			**1.601 (1.034–2.480)**	**0.035**		
AA (MR)			–	–		
CA (MOD)			0.698 (0.449–1.085)	0.110		
***SLC22A5:* rs274558**						
AA	64	32.3	Ref.		83	20.5
AG	101	51.0	0.668 (0.446–1.002)	0.051	196	48.5
GG	33	16.7	**0.342 (0.207–0.566)**	**<0.001**	125	30.9
AA (MD)			**1.847 (1.259–2.710)**	**0.002**		
GG (MR)			**0.446 (0.291–0.686)**	**<0.001**		
AG (MOD)			1.105 (0.786–1.553)	0.565		
***SLC22A5:* rs2631365**						
TT	67	33.8	Ref.		92	22.8
TC	101	51.0	**0.630 (0.425–0.934)**	**0.021**	220	54.4
CC	30	15.2	**0.448 (0.267–0.752)**	**0.002**	92	22.8
TT (MD)			**1.734 (1.192–2.524)**	**0.004**		
CC (MR)			**0.606 (0.385–0.952)**	**0.030**		
TC (MOD)			0.871 (0.620–1.224)	0.426		
**Number of risk genotypes**						
0	12	6.1	Ref.		56	13.9
1	89	44.9	**2.006 (1.025–3.926)**	**0.042**	207	51.2
2	58	29.3	**2.256 (1.122–4.533)**	**0.022**	120	29.7
3 or more	39	19.7	**8.667 (3.822–19.650)**	**<0.001**	21	5.2

The OR (95% CI) and *p* values were calculated by logistic regression according to the following genetic models: The codominant model (MCD)—where each genotype was compared with the homozygous major allele (mm or mM vs. MM); the dominant model (MD)—minor allele carriers against major allele homozygous (mm + mM vs. MM); the recessive model (MR)—minor allele homozygous compared with major alleles carriers (mm vs. MM + mM); and the overdominant model (MOD)—heterozygous against homozygous individuals (mM vs. MM + mm). For the number of risk genotypes analysis, *ABCB1* (rs1045642), *ABCG2* (rs2231142) and *SLC22A5* (rs274558 and rs2631365) were included. M: major allele; m: minor allele. OR: odds ratio; CI: confidence interval; CML: chronic myeloid leukaemia; Ref.: reference.

## Data Availability

The datasets used and/or analysed during the current study are available from the corresponding author on reasonable request.

## Supplementary Materials

The supporting information can be downloaded at: https://www.mdpi.com/article/10.3390/ijms23179815/s1.

## Abbreviations

ABC: ATP-binding cassette; *ABCB1*: *ATP binding cassette subfamily B member 1*; *ABCG2*: *ATP binding cassette subfamily G member 2*; *ABL1*: *ABL proto-oncogene 1, non-receptor tyrosine kinase*; *BCR: BCR activator of RhoGEF and GTPase*; BCRP: Breast cancer resistance protein; CI: Confidence interval; CML: Chronic myeloid leukaemia; ELN: European LeukemiaNet; GP: Genotypic profile; HR: Hazard ratio; HWE: Hardy-Weinberg equilibrium; MAF: Minor allele frequency; MCD: Codominant model; MD: Dominant model; MOD: Overdominant model; MR: Recessive model; OCT1: Organic cation transporter 1; OCTN2: Organic cation/carnitine transporter 2; OR: Odds ratio; P-gP: P-glycoprotein; SLC: Solute carrier; *SLC22A1: Solute carrier family 22 member 1*; *SLC22A5: Solute carrier family 22 member 5*; SNVs: Single nucleotide variants; TKIs: Tyrosine kinase inhibitors.
