# Supplementary material for: Genetic Variants of ABC and SLC Transporter Genes and Chronic Myeloid Leukaemia: Impact on Susceptibility and Prognosis
Source: Int J Mol Sci. 2022 Aug 29;23(17):9815. doi: 10.3390/ijms23179815 (PMC9456284; doi:10.3390/ijms23179815)
Supplement: Supplementary file 1 [file ijms-23-09815-s001.zip › ijms-1852648-supplementary.pdf]

Supplementary Material

Genetic variants of ABC and SLC transporter genes and chronic myeloid leukaemia: impact on susceptibility and prognosis

Raquel Alves [1,2,3], Ana Cristina Gonçalves [1,2,3*], Joana Jorge [1,2,3], Gilberto Marques [4], André B. Ribeiro [2,5], Rita Tenreiro [5], Margarida Coucelo [2,5], Joana Diamond [6], Bárbara Oliveiros [2,7], Amélia Pereira [2,8,9], Paulo Freitas-Tavares [5], António M. Almeida [10,11], and Ana Bela Sarmento-Ribeiro [1,2,3,5*]

Table S1. Characterisation of the 10 selected SNVs

Gene Symbol*	Drug Transporter	dbSNP	Chr. Position[†]	Variants	Molecular Consequence	Iberian MAF[‡]	Clinical significance[$]
ABCB1	Efflux	rs1045642	7:87509329	c.3435T>C	Synonymous	0.463 (T)	Drug-response allele
		rs1128503	7:87550285	c.1236T>C	Synonymous	0.378 (T)	Benign allele
		rs2032582	7:87531302	c.2677T>G<br>p.Ser893Ala	Missense	0.364 (T)	Drug-response
ABCG2	Efflux	rs2231142	4:88131171	c.421C>A<br>p.Gln141Lys	Missense	0.070 (A)	Drug-response allele
		rs2231137	4:88139962	c.34G>A<br>p. Val12Met	Missense	0.051 (A)	Affects, association
SLC22A1	Influx	rs628031	6:160139813	c.1222A>G<br>p. Met408Leu	Missense	0.429 (A)	Not Reported
		rs683369	6:160130172	c.480G>C<br>p.Leu160Phe	Missense	0.243 (G)	Not Reported
		rs1867351	6:160122091	c.156T>C	Synonymous	0.196 (C)	Not Reported
SLC22A5	Influx	rs274558	5:132385482	c.807A>G	Synonymous	0.364 (G)	Benign
		rs2631365	5:132370257	c.285T>C	Synonymous	0.369 (C)	Benign

*According to HUGO Gene Nomenclature Committee (HGNC). [†]Chromosome position based on GRCh38.p12. [‡]MAF, minor allele frequency according to the 1000 Genome database (Caucasians/European/Iberian population in Spain). [$]Clinical significance reported on ClinVar database. *ABCB1*: ATP binding cassette subfamily B member 1; *ABCG2:* ATP binding cassette subfamily G member 2; *SLC22A1*: solute carrier family 22 member 1; *SLC22A5*: solute carrier family 22 member 5.

Table S2. Tetra-primer-ARMS-PCR general conditions

Gene	dbSNP	Primers (5′→ 3′)	PCR Conditions	PCR Program	PCR Products
ABCB1	rs1045642	FO: GCTACATTCAAAGTGTGCTGGTCCTG<br>RO: TGGGAGACCAGCCCCTTATAAATCA<br>FI: GGGTGGTGTCACAGGAAGAGCTC<br>RI: CCTCCTTTGCTGCCCTCCCA	$MgCl_2$ 1.5 mM<br>FO/RO 100 nM<br>FI/RI 250 nM	30′′ 95°C<br>30′′ 62°C x35<br>30′′ 72°C	Control: 346 bp<br>Allele T: 226 bp<br>Allele C: 163 bp
	rs1128503	FO: TTCACTTCAGTTACCCATCTCGAA<br>RO: GTCTAGCTCGCATGGGTCATC<br>FI: CGTCCTGGTAGATCTTGAAGTGC<br>RI: CTGCACCTTCAGGTTCCGA	$MgCl_2$ 2.0 mM<br>FO/RO 150 nM<br>FI/RI 250 nM	30′′ 95°C<br>30′′ 59°C x35<br>30′′ 72°C	Control: 357 bp<br>Allele T: 236 bp<br>Allele C: 163 bp
	rs2032582	FO: CTAATTTGTTTTGTTTTGCAGGCTATAG<br>RO: TTACTGAATAAGCATGAAAAAGATTGCT<br>FI: CACTGAAAGATAAGAAAGAACTAGAAGATG<br>RI: ATATTTAGTTTGACTCACCTTCCCGGA	$MgCl_2$ 2.0 mM<br>FO/RO 150 nM<br>FI/RI 150 nM	30′′ 95°C<br>30′′ 58°C x35<br>30′′ 72°C	Control: 371 bp<br>Allele T: 243 bp<br>Allele G: 185 bp
ABCG2	rs2231142	FO: GGTGTACAGATAGGGGGTGAAAAA<br>RO: TTTTATCCACACAGGGAAAGTCCT<br>FI: CTCTGACGGTGAGAGAAAACTCAA<br>RI: CCGAAGAGCTGCTGAGAATTG	$MgCl_2$ 1.5 mM<br>FO/RO 100 nM<br>FI/RI 100 nM	30′′ 95°C<br>30′′ 59°C x35<br>30′′ 72°C	Control: 428 bp<br>Allele C: 274 bp<br>Allele A: 199 bp
	rs2231137	FO: CTTCCAGTAATGTCGAAGTTTTTATCCAAG<br>RO: AAGCCATTGGTGTTTCCTTGTGAAAT<br>FI: GTGCCTGTCTTCCCATTTAGGTTTTTAG<br>RI: CTTTCTCAACTGGTTTTCGACAAGGTAG	$MgCl_2$ 1.5 mM<br>FO/RI 500 nM<br>RO/FI 250 nM<br>DMSO 10%	30′′ 95°C<br>30′′ 54°C x35<br>30′′ 72°C	Control: 386 bp<br>Allele A: 261 bp<br>Allele G: 181 bp
SLC22A1	rs628031	FO: TGTGCTCTATCAGGGGCTCATCCT<br>RO: TCCCCACACTTCGATTGCCTG<br>FI: CCGCATCTACCCCATGGACG<br>RI: CCCCCGCCAACAAATTTGAAAT	$MgCl_2$ 3.0 mM<br>FO/RO 150 nM<br>FI/RI 250 nM<br>DMSO 10%	30′′ 95°C<br>30′′ 61°C x35<br>30′′ 72°C	Control: 258 bp<br>Allele A: 170 bp<br>Allele G: 130 bp
	rs683369	FO: GCTGAAGAGAGGATGGAAGGGTG<br>RO: CAAAGCAGGGGCCACTGTGT<br>FI: TGTTTGAATGCGGGCTTCATC<br>RI: GCCAACACCGAGAGAGCCATAC	$MgCl_2$ 1.5 mM<br>FO/RO 100 nM<br>FI/RI 250 nM	30′′ 95°C<br>30′′ 60°C x35<br>30′′ 72°C	Control: 337 bp<br>Allele C: 204 bp<br>Allele G: 176 bp
	rs1867351	FO: CTTCATAGCGCCTGCACTGGC<br>RO: ACGGCCATGAGCATGCTGAG<br>FI: CAGCTCAGCCACCCCATGG<br>RI: CTGACCACCACTGCCAGCGT	Buffer 1.5x<br>$MgCl_2$ 1.4 mM<br>FO/RI 150 nM<br>RO/FI 250 nM	30′′ 95°C<br>30′′ 60°C x35<br>30′′ 72°C	Control: 302 bp<br>Allele C: 200 bp<br>Allele T: 141 bp
SLC22A5	rs274558	FO: TTTTTCCCCACACTTGGGGC<br>RO: CGACCTCCCTTGTTTTGAACAGG<br>FI: CACCACCAGAGTGCCACGAAC<br>RI: GACGATGCCGGGGGTGATA	$MgCl_2$ 2.5 mM<br>FO/RI 300 nM<br>RO/FI 150 nM<br>DMSO 10%	30′′ 95°C<br>45′′ 51°C x35<br>60′′ 72°C	Control: 433 bp<br>Allele T: 275 bp<br>Allele C: 176 bp
	rs2631365	FO: TGACCGTTGGGGGAGGGGGA<br>RO: TAGCGACCCCGGAGCACCGC<br>FI: CGCCCCGGCTCCAGCACA<br>RI: ACCATCGCCAACTTCTCGGCGATC	$MgCl_2$ 2.0 mM<br>FO/RO 150 nM<br>FI/RI 250 nM<br>DMSO 10%	30′′ 95°C<br>30′′ 60°C x35<br>30′′ 72°C	Control: 389 bp<br>Allele G: 255 bp<br>Allele A: 176 bp

FO: forward outer primer; RO: reverse outer primer; FI: Forward inner primer; RI: reverse inner primer; $MgCl_2$: Magnesium chloride; DMSO: Dimethyl sulfoxide.
